# Impact of Cryotherapy on Sensory, Motor, and Autonomic Neuropathy in Breast Cancer Patients Receiving Paclitaxel: A Randomized, Controlled Trial

**DOI:** 10.3389/fneur.2020.604688

**Published:** 2020-12-18

**Authors:** Ding Quan Ng, Chia Jie Tan, Boon Chua Soh, Mabel May Leng Tan, Soon Yue Loh, Yam Eng Tan, Hui Hui Ong, Peggy Pei Chee Teng, Jack Junjie Chan, Wen Yee Chay, Joycelyn Lee, Gillianne Lai, Sok Yuen Beh, Tira Jing Ying Tan, Yoon Sim Yap, Guek Eng Lee, Mabel Wong, Rebecca Dent, Yew Long Lo, Alexandre Chan, Kiley Wei-Jen Loh

**Affiliations:** ^1^Department of Pharmacy, National University of Singapore, Singapore, Singapore; ^2^Department of Clinical Pharmacy Practice, University of California, Irvine, Irvine, CA, United States; ^3^Division of Medical Oncology, National Cancer Centre Singapore, Singapore, Singapore; ^4^Division of Nursing, National Cancer Centre Singapore, Singapore, Singapore; ^5^Department of Neurology, Singapore General Hospital, Singapore, Singapore; ^6^Duke-NUS Medical School, Singapore, Singapore; ^7^National Neuroscience Institute, Singapore, Singapore; ^8^Department of Pharmacy, National Cancer Centre Singapore, Singapore, Singapore

**Keywords:** breast cancer, paclitaxel, chemotherapy-induced peripheral neuropathy, cryotherapy, patient-reported outcome, electrophysiological assessment

## Abstract

**Introduction:** We conducted a randomized controlled trial evaluating the efficacy and tolerability of cryotherapy in preventing chemotherapy-induced peripheral neuropathy (CIPN) in patients with early breast cancer receiving neo/adjuvant weekly paclitaxel.

**Methods:** Patients were recruited from the National Cancer Centre Singapore and randomized (1:1) to receive either cryotherapy or usual care. Cryotherapy was applied as frozen gloves and socks on all extremities from 15 min before paclitaxel until 15 min post-infusion every cycle. Efficacy was measured by patient-reported outcomes (Patient Neurotoxicity Questionnaire [PNQ] and EORTC QLQ-CIPN20) and electrophysiological assessments. The primary endpoint was PNQ severity at 2 weeks after 12 cycles of weekly paclitaxel.

**Results:** A total of 46 patients were recruited, of which 8 dropped out before paclitaxel treatment, leaving 38 evaluable. There was no significant difference in PNQ severity between cryotherapy and usual care at 2 weeks after paclitaxel treatment (sensory: *p* = 0.721; motor: *p* = 1.000). A benefit was observed at 3 months post-paclitaxel based on PNQ (sensory: 14.3 vs. 41.2%, *p* = 0.078; motor: 0 vs. 29.4%, *p* = 0.012) and CIPN20 (sensory: β = −3.6, 95%CI = −10.5–3.4, *p* = 0.308; motor: β = −7.3, 95%CI = −14.6–0, *p* = 0.051). Additionally, cryotherapy subjects have lower CIPN20 autonomic score (β = −5.84, 95%CI = −11.15 to −0.524, *p* = 0.031) and higher sympathetic skin response hand amplitudes (β = 0.544, 95%CI = 0.108–0.98, *p* = 0.014), suggesting possible autonomic benefits from cryotherapy. Temporary interruption with cryotherapy occurred in 80.9% of the subjects due to cold intolerance.

**Conclusions:** There is insufficient evidence that cryotherapy prevents sensory neuropathy which may be due to the high rates of cryotherapy interruption in this study. The autonomic benefits of cryotherapy should be further investigated with appropriate outcome measures.

**Clinical Trial Registration:** ClinicalTrials.gov: NCT03429972.

## Introduction

Adjuvant systemic therapy has contributed to significant improvements in breast cancer survival globally (1). Of these, adjuvant chemotherapy decreases the risk of recurrence, breast cancer mortality and overall mortality in breast cancer patients (2). However, survivors of breast cancer often experience acute and chronic complications after chemotherapy and the resultant detriment to quality of life (QoL) cannot be ignored (3, 4). Paclitaxel, which is ubiquitously used in early and locally-advanced breast cancers, causes chemotherapy-induced peripheral neuropathy (CIPN) in up to 77% of cancer patients (5, 6).

Paclitaxel-induced peripheral neuropathy predominantly manifests as sensory symptoms, such as numbness and paresthesia in the extremities (7). These symptoms are associated with poorer QoL, hindering activities of daily living, such as dressing and walking (8). Such reduction in QoL could persist in the long term, with 44% of patients who were treated with paclitaxel continuing to experience sensory symptoms 2 years after diagnosis (9). Dose reductions, treatment delays and discontinuations related to unbearable CIPN symptoms also prevent patients from receiving treatments at optimal intensity, which may be associated with poorer outcomes (10). To date, high-quality and consistent evidence to support the recommendation of any preventive measures for CIPN is limited (11, 12). This inadequacy in evidence-based CIPN management protocol, coupled with the pervasiveness and consequences of CIPN in patients, represent an area of unmet clinical need.

Efforts to explore a suitable preventive measure have led to the use of regional cryotherapy. The first evidence came as an explorative analysis, revealing that patients utilizing frozen gloves and socks for the prevention of docetaxel-induced nail toxicity experienced a 44% reduction in the risk of CIPN (13). Other studies have examined if similar benefits can be observed among patients receiving chemotherapy (14–20). However, the findings of these studies have been inconsistent, exemplifying the need for more confirmatory studies (21).

We designed a randomized controlled trial to investigate the efficacy of cryotherapy, delivered using frozen gloves and socks, in preventing CIPN among early or locally advanced breast cancer patients receiving weekly paclitaxel based on patient-reported outcomes and objective electrophysiological assessments. We also aimed to describe the tolerance of cryotherapy among the study participants.

## Materials and Methods

### Study Design

This study is a prospective, parallel assignment, randomized controlled trial (ClinicalTrials.gov: NCT03429972), performed at the National Cancer Centre Singapore. Approval was obtained from SingHealth Centralized Institutional Review Board, Singapore (CIRB 2015/3017) and written informed consent were obtained from all participants.

### Participants

Potential participants were identified and referred by their primary oncologists. Participants fulfilled the following inclusion criteria: at least 21 years old, histologically confirmed early or locally advanced breast cancer, and scheduled to receive 12 cycles of weekly paclitaxel (80 mg/m^2^ infused over 60 min). Patients with a history of peripheral neuropathy, prior use of taxane-based chemotherapy and concurrent use of other neurotoxic chemotherapy were excluded. For safety reasons, patients with history of Raynaud's disease were ineligible. Participants with axillary clearance were required to install a central venous access device for chemotherapy administration to avoid peripheral cannulation obstruction during cryotherapy. Eligible patients were randomized 1:1 into control or cryotherapy groups. Block randomization was performed with a block size of 2. Investigators involved in recruitment were blinded to the randomization block size and the order of recruitment.

### Cryotherapy Intervention

Cryotherapy was delivered using Elasto-Gel™ hypothermia gloves and socks (−20 to −10°C) on both hands and feet in the intervention group. Participants wore the devices 15 min before the paclitaxel infusion until 15 min post-infusion, for a total of 90 min. The devices were timed to be changed every 30 (±5) min, to maintain optimal vasoconstrictive hypothermic conditions. Additionally, participants wore disposable polyethylene gloves and polypropylene sock liners inside the gloves and socks, respectively for hygiene purposes. During cryotherapy administration, periods of temporary interruption were allowed for cryotherapy-intolerant participants.

### Outcome Measures

**Figure 1:**
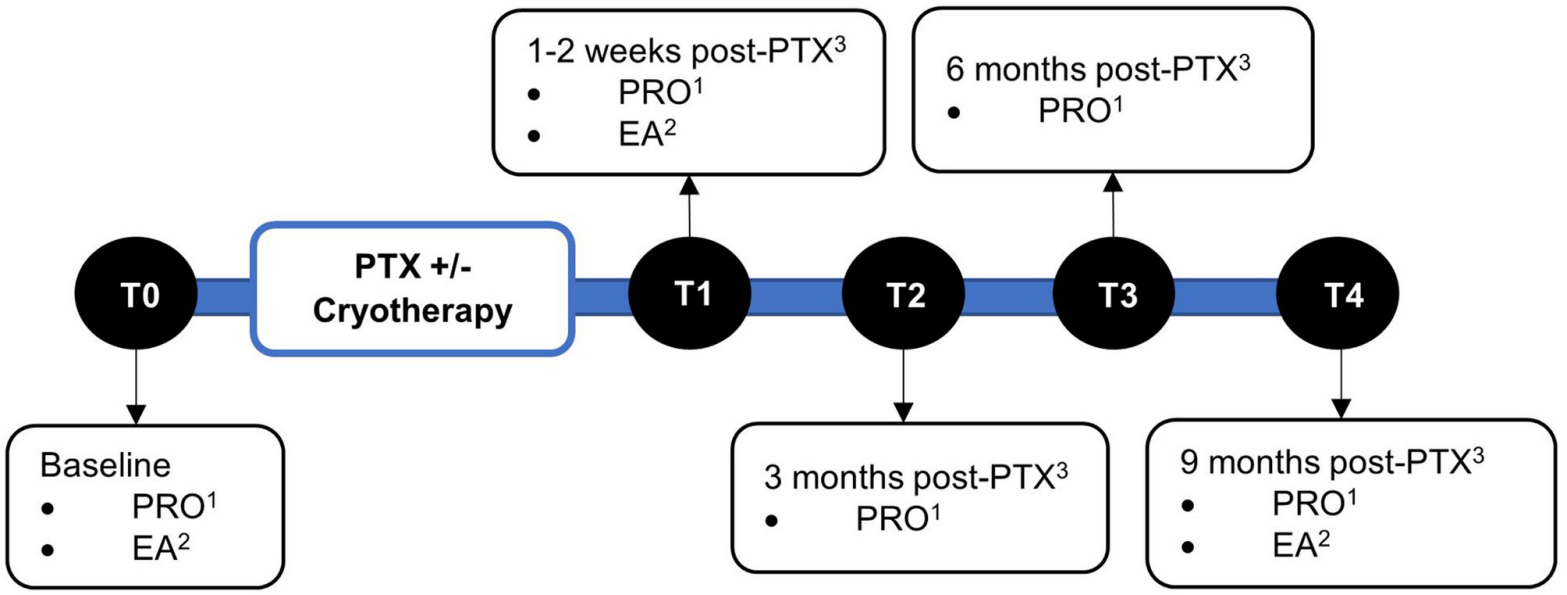
Timeline of assessments. Assessments were performed at baseline (T0), 1–2 weeks (T1), 3 months (T2), 6 months (T3), and 9 months (T4) post-completion of paclitaxel treatment. ^1^Patient-reported outcomes. ^2^Electrophysiological assessments. ^3^Paclitaxel.

Baseline demographic and clinical parameters were retrieved from electronic medical records. The efficacy of cryotherapy was evaluated using patient-reported outcome (PRO) questionnaires and electrophysiological assessments. PRO questionnaires were administered at baseline prior to paclitaxel, 1–2 weeks, 3-, 6-, and 9-months post-paclitaxel treatment (T0–T4). Electrophysiological assessments were conducted at baseline, 1–2 weeks, and 6 months post-paclitaxel treatment (T0, T1, and T3, respectively) (Figure 1).

#### Patient-Reported Outcome (PRO) Questionnaires

Sensory and motor CIPN symptoms were assessed with Patient Neurotoxicity Questionnaire (PNQ) and EORTC QLQ-CIPN twenty-item scale (CIPN20). Autonomic symptoms were examined with CIPN20. The sum score of eighteen CIPN20 items, excluding conditional items 19 and 20, represent the degree of CIPN symptom burden (22, 23). Clinically important symptoms were determined as PNQ grades C-E as they delineate moderate to severe CIPN symptoms. For CIPN20, higher scores indicate a greater severity of symptoms (22, 23).

The EORTC Quality of Life Questionnaire-Core 30 (version 3) (C30) was employed to evaluate health-related QoL (HRQoL). C30 subscales for global health status (GHS), physical (PF), and role functioning (RF), and pain symptoms were examined. Higher scores represent a better global health status and better degree of functioning while lower pain scores indicate less severe pain (24). Detailed descriptions of these PRO questionnaires can be found in the Supplementary Material.

#### Electrophysiological Assessments

Nerve conduction study (NCS) and sympathetic skin response (SSR) were performed by two designated technologists using a Dantec Counterpoint EMG machine (Dantec, Skovlunde, Denmark). In this study, higher amplitudes and conduction velocities, and lower onset latencies are defined as preserved nerve function (25, 26).

##### 1.Nerve conduction study (NCS)

NCS is an objective measure used to characterize nerve dysfunction in patients (27). We performed NCS using adhesive surface recording electrodes and hand-held stimulating cathode placed proximally. All patients were in their supine position in a quiet room. A total of 10 averages were captured to obtain an optimal sensory nerve action potential (SNAP). For motor studies, supramaximal stimulation ensured that the highest compound motor action potential (CMAP) amplitudes were elicited. Onset latencies, conduction velocities, peak to peak SNAP amplitudes, and baseline to peak CMAP amplitudes were obtained with amplifier filter bands ranging from 20 Hz to 2 kHz, and surface skin temperature at or above 33°C. Median, ulnar, radial, peroneal and sural sensory nerves, as well as median, ulnar, tibial, and peroneal motor nerves of both limbs were assessed. Amplitudes of sural SNAP and peroneal CMAP were recognized as they were highly correlated to the development of sensory and motor peripheral neuropathy, respectively (28).

##### 2.Sympathetic skin response (SSR)

SSR serves as a marker of small fiber neuropathy as it could detect anomalies in autonomic efferent fibers, and long-term denervation of sweat glands and afferent sensory fibers (26). Similar to NCS, surface recording electrodes were used, with the active electrode placed on the palmar or plantar surfaces of all four limbs, under the same environmental conditions. The reference electrodes were placed on the dorsum of the hand or foot. Stimulation was achieved electrically with a 0.2 ms current up to 40 mA at the wrist (median nerve) or behind the medial malleolus (posterior tibial nerve). Each stimulation was separated by at least 2 min to avoid habituation. Repeat stimulations were made to ensure reproducibility before a response was graded as present or absent. A ground electrode was placed proximal to the recording electrode on the limb studied. Baseline to negative peak amplitude and onset latencies were measured in all responses. The amplifier filter settings ranged from 0.2 to 100 Hz. SSR amplitudes, onset latencies and absent responses were analyzed for both hands and feet of the subjects.

#### Tolerance

Frequencies and reasons of device removal throughout the study were documented every cryotherapy cycle.

### Endpoints

The primary endpoint was the proportion of participants reporting PNQ grade C-E symptoms at 1–2 weeks after completing 12 cycles of weekly paclitaxel (T1), when CIPN prevalence peaks (29). For PRO, secondary endpoints include the differences in proportions of PNQ grade C-E symptoms at 3-, 6-, and 9-months post-paclitaxel (T2–T4, respectively), CIPN20 sensory, motor, autonomic and sum scores, and C30 GHS, PF, RF, and pain scores at the same four timepoints. Other secondary endpoints included longitudinal changes in PNQ and CIPN20-graded symptoms, C30 subscales, electrophysiological parameters, and cryotherapy tolerance.

### Statistical Analyses

Based on a postulated effect size of 48% for the primary endpoint (30), 5% significance level, 80% power, and an assumed dropout rate of 20% for both groups, a total of 46 participants is required. Sample size was calculated using Power and Sample Size Calculation version 3.1.6 (31).

Continuous data is summarized with means and standard deviations (SD), or medians and interquartile ranges (IQR) depending on skewness, while categorical data is presented as counts (*n*) and percentages. For cross-sectional analyses, either Chi-square test or Fisher's exact test was performed to compare proportions of PNQ-graded symptoms and absent SSR responses. Effects of cryotherapy on CIPN20 and C30 domain scores are analyzed by linear regression. Electrophysiological data was analyzed by mixed effects models adjusted for baseline values, with random intercepts for individual participants to handle correlation of left and right limb parameters. An absence of response was substituted with zero in amplitudes and conduction velocities. Onset latencies underwent reciprocal transformation so that absent responses can be imputed with zeroes as well.

To analyze longitudinal changes, mixed effects models adjusted for time (treated as a continuous variable) and baseline values and with random intercepts for individual participants were generated. For electrophysiological parameters, an additional level that grouped the right and left limb parameters recorded at the same visit were included.

All statistical tests were two-sided and *p* < 0.05 was considered statistically significant. Stata/SE version 16.0 was used to execute all statistical analyses.

## Results

### Participants

**Figure 2:**
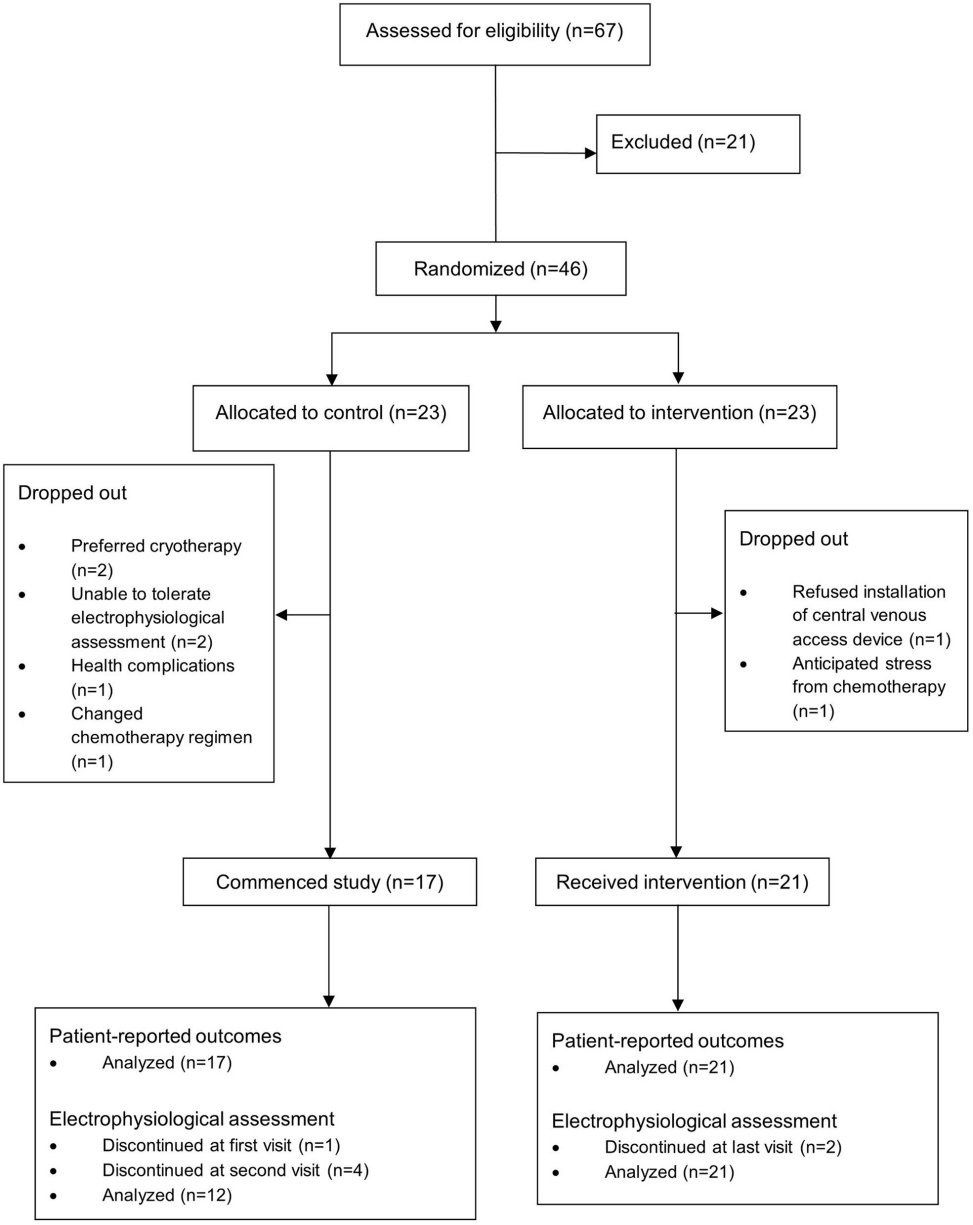
CONSORT Diagram.

Between April 2017 to December 2018, a total of 46 participants were recruited, of which 8 dropped out before receiving treatment, leaving 38 for modified intention-to-treat (mITT) analysis. One participant did not complete cryotherapy due to discontinuation of treatment (Figure 2). Twelve control and 21 cryotherapy participants completed electrophysiological assessment for at least three timepoints and were included in the analysis (Figure 2).

The mean age was 53.6 (SD: 7.6) years in the control group and 56.3 (SD: 8.1) years in the cryotherapy group. Cumulative paclitaxel dose was comparable, at 929.4 (SD: 28.6) mg/m^2^ in the control group and 925.3 (SD: 57.5) mg/m^2^ among cryotherapy participants (Table 1).

**Table 1:** Demographics and clinical characteristics of participants.

**Characteristics**	**Control**	**Cryotherapy**
	**(*N* = 17)**	**(*N* = 21)**
**DEMOGRAPHIC CHARACTERISTICS**		
Age in years, mean (SD)	53.6 (7.6)	56.4 (8.1)
Ethnicity, *n* (%)		
Chinese	14 (82.4)	16 (76.2)
Malay	2 (11.8)	4 (19)
Indian	1 (5.9)	0 (0)
Others	0 (0)	1 (4.8)
ECOG^a^, *n* (%)		
0	16 (94.1)	18 (85.7)
1	1 (5.9)	3 (14.3)
Diabetic, *n* (%)	1 (5.9)	3 (14.3)
**CHEMOTHERAPY**		
PTX^b^ cumulative dose in mg/m^2^, mean (SD)	929.4 (28.6)	925.3 (57.5)
PTX^b^ treatment type, *n* (%)		
Adjuvant	8 (47.1)	13 (61.9)
Neo-adjuvant	9 (52.9)	8 (38.1)
Exposure to AC^c^, *n* (%)		
Prior to PTX^b^	13 (76.5)	17 (81)
After PTX^b^	0 (0)	1 (4.8)
No exposure	4 (23.5)	3 (14.3)
**CLINICAL CHARACTERISTICS**		
Breast cancer stage, *n* (%)		
Stage 1	4 (23.5)	4 (19)
Stage 2	8 (47.1)	10 (47.6)
Stage 3	5 (29.4)	7 (33.3)
Cancer histology, *n* (%)		
ER/PR positive	15 (88.2)	13 (61.9)
HER2 positive	9 (52.9)	11 (52.4)
Other treatment modalities, *n* (%)		
Targeted therapy	9 (52.9)	11 (52.4)
Radiotherapy	12 (70.6)	17 (81)
Endocrine therapy	13 (76.5)	13 (61.9)
**BASELINE PATIENT-REPORTED OUTCOMES**		
PNQ^d^, Sensory, *n* (%)		
A, B	17 (100)	21 (100)
C, D, E	0 (0)	0 (0)
PNQ^d^, Motor, *n* (%)		
A, B	17 (100)	20 (95.2)
C, D, E	0 (0)	1 (4.8)
CIPN20^e^ Scores, mean (SD)		
Sensory	2.2 (4.2)	1.8 (3.6)
Motor	2.5 (3.8)	3.9 (6.1)
Autonomic	9.8 (11.9)	9.5 (11.3)
Sum	3.2 (3.6)	3.4 (3.9)
**BASELINE ELECTROPHYSIOLOGICAL PARAMETERS**		
Sural SNAP^f^ amplitude in μV, mean (SD)	14.3 (7)	12.6 (6.7)
Peroneal CMAP^g^ amplitude in mV, mean (SD)	4.4 (1.2)	4.3 (2)
SSR^h^ absent responses, *n* (%)		
Hands	2 (12.5)	1 (4.8)
Feet	1 (6.3)	1 (4.8)

a *ECOG, Eastern Cooperative Oncology Group Performance Status*.

b *PTX, paclitaxel*.

c *AC, anthracycline-cyclophosphamide*.

d *PNQ, Patient Neurotoxicity Questionnaire. CIPN symptom severity is graded from A (no neuropathy) to E (severe neuropathy) on a Likert scale*.

e *CIPN20, European Organisation for Research and Treatment of Cancer Quality of Life Questionnaire-CIPN twenty-item scale. Lower CIPN20 sum score represents a lower degree of CIPN symptom burden*.

f *SNAP, sensory nerve action potential. Higher SNAP amplitude represents more functional sensory nerve fibers*.

g *CMAP, compound motor action potential. Higher CMAP amplitude represents more functional motor nerve and muscle fibers*.

h *SSR, sympathetic skin response. An absent SSR response in at least one limb represents a relatively severe degree of autonomic dysfunction*.

### Sensory Symptoms

**Figure 3:**
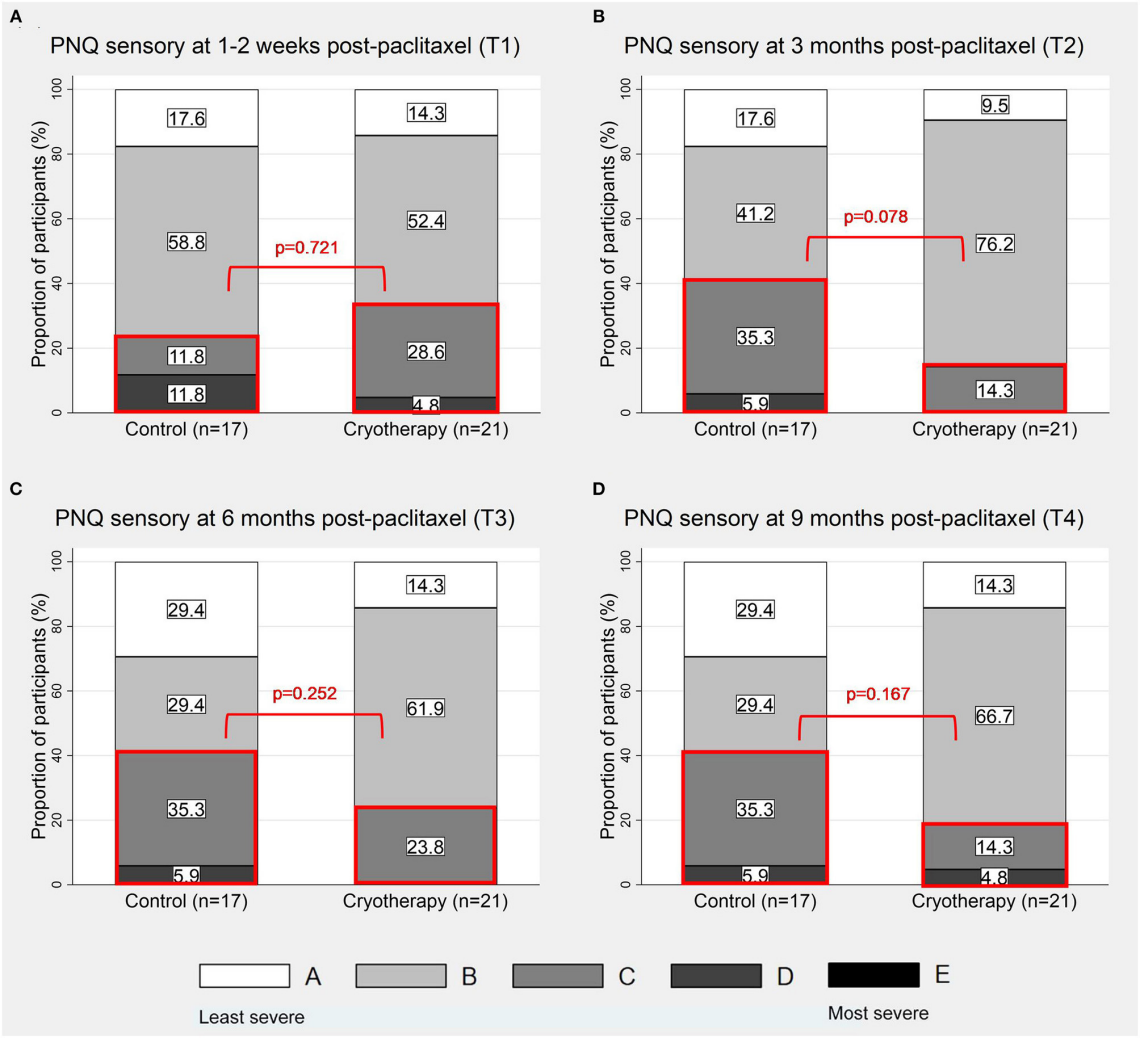
Proportion of participants with PNQ grades A-E sensory symptoms. The figures show the distribution of PNQ-graded sensory symptoms at 1–2 weeks **(A)**, 3 months **(B)**, 6 months **(C)**, and 9 months **(D)** post-completion of paclitaxel. The sections of the graphs which were in red boxes represent the proportions of participants with PNQ grades C-E symptoms.

At T1, 33.3% (7/21) of participants in the cryotherapy group reported PNQ grades C-E sensory symptoms compared to 23.5% (4/17) in the control group (*p* = 0.721, Figure 3A), thus the primary endpoint was not met. No difference was observed at this timepoint with CIPN20 (β = 2.992, 95%CI = −4.698–10.68, *p* = 0.435, Table 2) and sural SNAP parameters (amplitude: β = −0.164, 95%CI = −3–2.672, *p* = 0.910; conduction velocity: β = 0.575, 95%CI = −8.671–9.821, *p* = 0.903; Table 4). Statistical significance was not reached at all other timepoints (Figures 3B–D; Tables 2, 4). Cross-sectionally, it was observed that the lowest incidence and severity of sensory symptoms occurred at T2 as graded by PNQ (Figure 3B) and CIPN20 (Table 2). Longitudinal analyses of PNQ, CIPN20 and sural SNAP parameters did not identify any benefits with cryotherapy at preventing or ameliorating sensory symptoms (Tables 3, 5). Analyses of other sensory nerves can be found in Supplementary Tables 1, 2.

**Table 2:** Linear regression analyses of EORTC QLQ-CIPN20 and C30 scores (*N* = 38).

	**1–2 weeks post-paclitaxel**			**3 months post-paclitaxel**			**6 months post-paclitaxel**			**9 months post-paclitaxel**		
	**(T1)**			**(T2)**			**(T3)**			**(T4)**		
	**β**	**95% CI**	***p*-value**	**β**	**95% CI**	***p*-value**	**β**	**95% CI**	***p*-value**	**β**	**95% CI**	***p*-value**
**CIPN20**												
Sensory	2.992	−4.698–10.68	0.435	−3.558	−10.54–3.423	0.308	1.878	−6.062–9.818	0.634	0.996	−8.484–10.48	0.832
Motor	−1.016	−7.849–5.817	0.765	−7.256	−14.556–0.044	0.051	−1.787	−9.47–5.896	0.640	−2.708	−9.979–4.564	0.455
Autonomic	−6.961	−16.2–2.275	0.135	−5.415	−13.32–2.486	0.173	−9.384	−16.76 to −2.009	0.014^*^	−3.454	−10.1–3.19	0.299
Sum	0.495	−6.157–7.147	0.881	−5.203	−11.11–0.706	0.083	−0.799	−7.352–5.754	0.806	−0.939	−8.245–6.367	0.796
**C30**												
GHS^a^	7.096	−3.977–18.17	0.202	3.828	−5.769–13.43	0.424	7.493	−0.695–15.68	0.072	10.691	1.5–19.88	0.024^*^
PF^b^	−2.222	−10.3–5.854	0.580	4.725	−3.127–12.58	0.230	2.988	−5.385–11.36	0.474	0	−8.054–8.054	1.000
RF^c^	0.373	−12.57–13.32	0.954	1.401	−12.54–15.34	0.840	0.047	−11.7–11.8	0.994	−2.521	−14.73–9.685	0.678
Pain	8.497	−5.151–22.15	0.215	−0.373	−13.05–12.3	0.953	1.261	−12.62–15.14	0.855	7.143	−6.001–20.29	0.278

*Positive βs for GHS, PF and RF imply that cryotherapy has led to better quality of life and functioning. Negative βs for CIPN20 scores and pain indicate that patients receiving cryotherapy have less severe symptoms*.

* *p < 0.05*.

a *Global Health Status*.

b *Physical Functioning*.

c *Role Functioning*.

**Table 3:** Mixed (logit) model analyses of changes in PNQ, EORTC QLQ-CIPN20 and C30 scores overtime, with baseline adjustment (*N* = 38).

	**Cryotherapy**			**Weeks (from baseline)**		
	**OR/β**	**95% CI**	***p*-Value**	**OR/β**	**95% CI**	***p*-Value**
**PNQ (≥grade C)**						
Sensory	0.649^#^	0.107–3.921	0.638	1.053^#^	1.029–1.076	<0.001^*^
Motor	0.451	0.079–2.565	0.369	1.005	0.98–1.032	0.687
**CIPN20**						
Sensory	0.688	−4.618–5.994	0.799	0.107	0.056–0.158	<0.001^*^
Motor	−3.115	−7.439–1.209	0.158	0.054	0.009–0.099	0.018^*^
Autonomic	−5.84	−11.15– −0.524	0.031^*^	−0.152	−0.215 to −0.089	<0.001^*^
Sum	−1.41	−5.719–2.899	0.521	0.057	0.017–0.097	0.006^*^
**C30**						
GHS^a^	3.157	−1.945–8.259	0.225	0.225	0.143–0.307	<0.001^*^
PF^b^	0.73	−3.538–4.998	0.737	−0.031	−0.088–0.025	0.276
RF^c^	0.962	−6.709–8.633	0.806	0.058	−0.033–0.149	0.211
Pain	4.773	−2.996–12.542	0.229	0.121	0.028–0.215	0.011^*^

*OR <1 for PNQ indicates lower odds of reporting neuropathy symptoms. Positive βs for GHS, PF and RF imply that cryotherapy has led to better quality of life and functioning. Negative βs for CIPN20 scores and pain indicate that patients receiving cryotherapy have less severe symptoms*.

* *p < 0.05*.

# *Baseline omitted due to collinearity*.

a *Global Health Status*.

b *Physical Functioning*.

c *Role Functioning*.

**Table 4:** Mixed model analyses of sural SNAP and peroneal CMAP parameters at 1–2 weeks and 6 months post-paclitaxel treatment, with baseline adjustment (*N* = 33).

	**Amplitude**						**Conduction velocity**					
	**1–2 weeks post-paclitaxel (T1)**			**6 months post-paclitaxel (T3)**			**1–2 weeks post-paclitaxel (T1)**			**6 months post-paclitaxel (T3)**		
	**β**	**95% CI**	***p*-value**	**β**	**95% CI**	***p*-value**	**β**	**95% CI**	***p*-value**	**β**	**95% CI**	***p*-value**
Sural SNAP^a^	−0.164	−3–2.672	0.910	−1.037	−2.632–0.557	0.202	0.575	−8.671–9.821	0.903	−6.51	−14.93–1.913	0.130
Peroneal CMAP^b^	0.34	−0.295–0.974	0.294	0.269	−0.207–0.744	0.269	0.173	−1.733–2.079	0.859	−1.552	−3.022 to −0.083	0.038^*^

*Positive β values indicate better nerve function*.

* *p < 0.05*.

a *SNAP, sensory nerve action potential*.

b *CMAP, compound motor action potential*.

**Table 5:** Mixed model analyses of changes in sural SNAP and peroneal CMAP parameters overtime, with baseline adjustment (*N* = 33).

	**Amplitude**						**Conduction velocity**					
	**Cryotherapy**			**Weeks (from baseline)**			**Cryotherapy**			**Weeks (from baseline)**		
	**β**	**95% CI**	***p*-value**	**β**	**95% CI**	***p*-value**	**β**	**95% CI**	***p*-value**	**β**	**95% CI**	***p*-value**
Sural SNAP^a^	−0.783	−2.94–1.373	0.477	−0.101	−0.139 – −0.063	<0.001^*^	−3.037	−8.824–2.75	0.304	−0.063	−0.164–0.037	0.217
Peroneal CMAP^b^	0.375	−0.137–0.887	0.151	0.001	−0.008–0.01	0.829	−0.754	−2.105–0.596	0.273	−0.018	−0.044–0.009	0.193

*Positive β values indicate better nerve function*.

* *p <0.05*.

a *SNAP, sensory nerve action potential*.

b *CMAP, compound motor action potential*.

### Motor Symptoms

**Figure 4:**
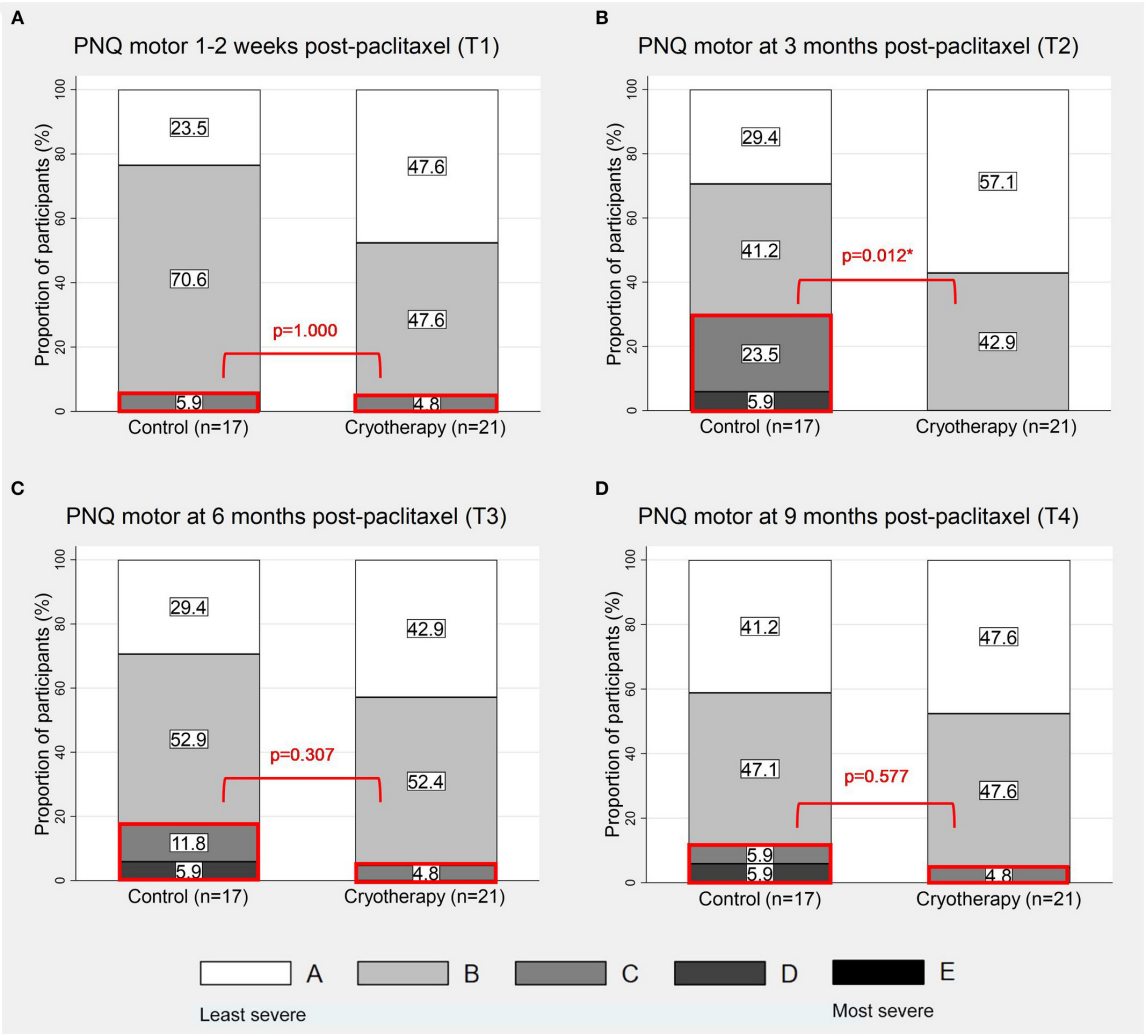
Proportion of participants with PNQ grades A-E motor symptoms. The figures show the distribution of PNQ-graded motor symptoms at 1–2 weeks **(A)**, 3 months **(B)**, 6 months **(C)**, and 9 months **(D)** post-completion of paclitaxel respectively. The sections of the graphs which were in red boxes represent the proportions of participants with PNQ grades C-E symptoms. *p < 0.05.

At T1, there is no difference in the incidence of participants reporting PNQ grades C-E motor symptoms (4.8 vs. 5.9%, *p* = 1.000, Figure 4A), in the severity of CIPN20-graded motor symptoms (β = −1.016, 95%CI = −7.849–5.817, *p* = 0.765, Table 2), and in the motor nerve function as assessed by peroneal CMAP parameters (amplitude: β = 0.34, 95%CI = −0.295–0.974, *p* = 0.294; conduction velocity: β = 0.173, 95%CI = −1.733–2.079, *p* = 0.859; Table 4). Similar to sensory symptoms, the greatest benefit was observed at T2, achieving statistical significance for PNQ (0 vs. 29.4%, *p* = 0.012, Figure 4B), and near significance for CIPN20 (β = −7.3, 95%CI = −14.6–0, *p* = 0.051, Table 2). However, cryotherapy subjects have statistically lower peroneal CMAP conduction velocity at T3 (β = −1.552, 95%CI = −3.022 to −0.083, *p* = 0.038, Table 4). Statistically significant difference was not observed at other timepoints (Figures 4C,D; Tables 2, 4). There is insufficient evidence to show the benefit of cryotherapy in preventing motor symptoms in the longitudinal analyses of PNQ, CIPN20, and peroneal CMAP data (Tables 3, 5). Analyses of other motor nerves can be found in Supplementary Tables 1, 2.

### Autonomic Function

No difference in the severity of autonomic symptoms, as graded by the CIPN20 autonomic subscale, was observed at T1 (β = −6.961, 95%CI = −16.2–2.275, *p* = 0.135, Table 2), although cryotherapy was shown to significantly reduce the severity at T3 (β = −9.384, 95%CI = −16.759 to −2.009, *p* = 0.014, Table 2). Moreover, cryotherapy subjects had significantly higher SSR amplitude in the hands compared to control subjects at T1 (β = 0.605, 95%CI = 0.047–1.164, *p* = 0.034, Table 6). The proportion of absent SSR responses were comparable at baseline (Table 1). However, the proportion at T1 increased from baseline and became much larger among control participants compared to cryotherapy subjects (hands: 19 vs. 41.7%, *p* = 0.230; feet: 14.3 vs. 41.7%, *p* = 0.106; Table 7) before decreasing to comparable levels at T3 (Table 7). Longitudinally, cryotherapy led to significantly lower CIPN20 autonomic score (β = −5.84, 95%CI = −11.15 to −0.524, *p* = 0.031, Table 3) and higher SSR hand amplitudes (β = 0.544, 95% CI = 0.108–0.98, *p* = 0.014, Table 8), but no effect was observed in SSR feet amplitudes (β = 0.305, 95%CI = −0.003–0.613, *p* = 0.053, Table 8). While statistical significance was not reached, there was a trend toward lower odds of developing absent SSR responses among cryotherapy patients in both hands and feet (hands: OR = 0.44, 95%CI = 0.08–2.434, *p* = 0.347; feet: OR = 0.232, 95%CI = 0.039–1.388, *p* = 0.109; Table 8).

**Table 6:** Mixed model analyses of SSR parameters at 1–2 weeks and 6 months post-paclitaxel treatment, with baseline adjustment (*N* = 33).

	**Amplitude**						**Reciprocal onset latency**					
	**1–2 weeks post-paclitaxel (T1)**			**6 months post-paclitaxel (T3)**			**1–2 weeks post-paclitaxel (T1)**			**6 months post-paclitaxel (T3)**		
	**β**	**95% CI**	***p*-value**	**β**	**95% CI**	***p*-value**	**β**	**95% CI**	***p*-value**	**β**	**95% CI**	***p*-value**
Hands	0.605	0.047–1.164	0.034^*^	0.524	−0.298–1.346	0.211	0.081	−0.105–0.268	0.393	0.051	−0.171–0.273	0.652
Feet	0.176	−0.098–0.45	0.209	0.33	−0.09–0.75	0.124	0.11	−0.038–0.258	0.144	0.005	−0.141–0.151	0.944

*Positive β values indicate better nerve function*.

* *p < 0.05*.

**Table 7:** Proportion of participants with absent SSR responses.

	**1–2 weeks post-paclitaxel (T1)**			**6 months post-paclitaxel (T3)**		
	**Control (*****n*** **=** **12)**	**Cryotherapy (*****n*** **=** **21)**	***p*****-value**	**Control (*****n*** **=** **12)**	**Cryotherapy (*****n*** **=** **19)**	***p*****-value**
Hands	5 (41.7)	4 (19)	0.230	3 (25)	4 (21.1)	1.000
Feet	5 (41.7)	3 (14.3)	0.106	3 (25)	4 (21.1)	1.000

*An absent SSR response in at least one limb represents a relatively severe degree of autonomic dysfunction. Data is presented as counts and percentages*.

**Table 8:** Mixed (logit) model analyses of changes in absent SSR responses, and SSR parameters overtime, with baseline adjustment (*N* = 33).

	**Cryotherapy**			**Weeks (from baseline)**		
	**OR/β**	**95% CI**	***p*-value**	**OR/β**	**95% CI**	***p*-value**
**HANDS**						
Absent SSR responses	0.44	0.08–2.434	0.347	1.02	0.981–1.061	0.309
Amplitude	0.544	0.108–0.98	0.014^*^	−0.003	−0.013–0.007	0.569
Reciprocal onset latency	0.104	−0.045–0.253	0.171	−0.001	−0.004–0.002	0.378
**FEET**						
Absent SSR responses	0.232	0.039–1.388	0.230	1.025	0.984–1.067	0.232
Amplitude	0.305	−0.003–0.613	0.053	0	−0.007–0.006	0.887
Reciprocal onset latency	0.065	−0.028–0.157	0.170	−0.001	−0.003–0.001	0.309

*OR <1 indicates lower odds of abnormal SSR response. Positive β values for amplitude and reciprocal onset latency indicate better nerve function. An absent SSR response in at least one limb represents a relatively severe degree of autonomic dysfunction*.

* *p < 0.05*.

### CIPN Symptom Burden

The CIPN symptom burden as rated using CIPN20 sum score was not different between cryotherapy and control groups cross-sectionally at each timepoint (Table 2), and longitudinally across all timepoints (β = −1.41, 95%CI = −5.719 to −2.899, *p* = 0.521, Table 3).

### Health-Related Quality of Life (HRQoL)

Significant difference favoring cryotherapy was observed for GHS at T4 (β = 10.691, 95%CI = 1.5–19.881, *p* = 0.024, Table 2), implying better overall quality of life among patients who had received cryotherapy at 9 months post-paclitaxel treatment. No difference was observed for PF, RF, and pain subscale scores at all recorded timepoints (Table 2). Comparable to PNQ and CIPN20, the greatest difference in PF and RF scores between the groups, favoring cryotherapy, was observed at T2 (Table 2). Mixed effects model analyses of GHS, PF, RF, and pain C30 subscale scores revealed no difference between cryotherapy and control participants (Table 3).

### Tolerance

For the average frequencies of device removal per cycle over the entire course of chemotherapy for each patient, the median was 0.958 (range: 0.125–5.5) for hands and 0.5 (range: 0–1.864) for feet. Restroom breaks (57.8%) and cryotherapy intolerance (36.5%) were the most common reasons for device removal. 17/21 (80.9%) subjects required temporary interruption of cryotherapy at least once during the entire course of chemotherapy due to cold intolerance. No other localized or severe adverse events (such as frostbites) secondary to cryotherapy were observed.

## Discussion

This study was a randomized controlled trial which investigated the effect of cryotherapy administered on all four limbs during paclitaxel treatment among early and locally advanced breast cancer patients. The objectives were to determine the efficacy and tolerability of cryotherapy in the prevention of paclitaxel-induced peripheral neuropathy using both PRO and objective electrophysiological assessments. Based on PRO results, cryotherapy did not lead to significantly lower incidence and severity of sensory symptoms. Similarly, the NCS findings did not report long-term improvements in sensory nerve functions among cryotherapy patients. Although we observed a lower incidence and severity of motor symptoms at 3 months post-paclitaxel treatment with PRO, its clinical relevance was limited as the benefits were not sustained in the long term. Moreover, motor CIPN symptoms, such as muscle weakness and cramps occurred less frequently than sensory symptoms among patients who have received paclitaxel (32–34). While cryotherapy patients reported better overall quality of life at 9 months post-paclitaxel, it should be interpreted with caution as this was not correlated to an improvement in neuropathic symptoms. Longitudinally, cryotherapy patients did not report better HRQoL than control subjects. In all, there is insufficient evidence supporting the use of cryotherapy in preventing sensory CIPN symptoms.

Interestingly, our SSR results demonstrated that cryotherapy may prevent small fiber neuropathy as interpreted from higher hand amplitudes among cryotherapy subjects. Consistency in favoring cryotherapy was observed for other SSR variables, albeit without statistical significance. Among paclitaxel-treated patients, small fiber neuropathy presents as autonomic dysfunction, paclitaxel acute pain syndrome (PAPS) and chronic neuropathic pain (35). Our cryotherapy subjects had better autonomic function according to the CIPN20 autonomic score, a composite of postural dizziness and blurred vision. One study found the association between cryotherapy and lower CIPN20 autonomic scores, although they concluded that clinical relevance was unlikely (19). Autonomic symptoms were infrequently reported and rarely considered cardinal adverse effects of paclitaxel (36, 37). Potentially, the observed improvement in SSR and CIPN20 autonomic subscale could be the inherent effect of cryotherapy, regardless of chemotherapy. The acute modulation of the autonomic nervous system follows the administration of cryotherapy (38, 39), although it is not known whether this effect is maintained in the long term. Cross-sectional findings based on CIPN20 and SSR were inconsistent, as statistical significance for CIPN20 autonomic score was observed at T3 relative to SSR which was observed at T1 instead. With distinct autonomic mechanisms underlying cardiovascular, ocular and sudomotor function (40), the effect of cryotherapy may differ for autonomic symptoms although the exact mechanism remains to be elucidated. Arguably, neither CIPN20 nor SSR are validated tools for measuring autonomic functions among paclitaxel-treated patients (41, 42). Additionally, the effect of cryotherapy on SSR hand parameters were not replicated in the feet. Thus, further validation is required with the use of more appropriate measures of autonomic functions, such as standing balance tasks, timed walking trials, and measuring variations in cardiac rhythms and blood pressure in response to changes in body posture (41, 43).

Our cryotherapy subjects did not report less pain according to our PRO results, although one study revealed that patients administered with frozen gloves complained of less aching or burning pain in the upper extremities (19). While less PAPS was not demonstrated in another study, cryotherapy was delivered differently using crushed ice instead of frozen gloves and socks (18). Recent evidence remained inconclusive about the effect of cryotherapy on pain. As neuropathic pain persists as a function-limiting symptom for cancer patients, future cryotherapy studies should continue gathering evidence on its efficacy with more elaborate and specific measures for neuropathic pain including quantitative sensory testing (44), and PRO, such as the Neuropathic Pain Symptom Inventory (NPSI) (45) or the Neuropathic Pain Scale for chemotherapy-induced neuropathy (NPS-CIN) (35, 46, 47). Regardless, due to the high dropout rates of control participants for electrophysiological assessments, our SSR results should be perceived as preliminary findings that warrant further investigation.

Existing randomized controlled trials that utilized the same Elasto-Gel™ hypothermia devices reported potential reduction in neuropathic symptoms with cryotherapy (19, 20). Less sensory and motor symptoms with cryotherapy was observed in one Japanese study (20), and less neuropathic symptoms leading to improved quality of life was observed in another study (19). Compared to our study, there are two possible explanations to delineate these differences. Firstly, by permitting periods of temporary cryotherapy interruption for intolerant patients, all subjects in our study had removed their devices at least once regardless of reasons. In comparison, the Japanese study reported that 68% of their patients were able to wear the hypothermia gloves and socks without interruption, and further analysis revealed greater cryotherapy benefits among these patients (20). This suggests that the continuous application of cryotherapy may be critical to produce a sustained benefit in preventing CIPN. It could be, however, challenging to emulate such a high degree of compliance in routine clinical practice, an observation that has been consistently reported across multiple studies (16, 19). Therefore, future studies should research the importance of variables, such as device storage temperature, skin temperature, skin blood flow, and duration of overall and continuous cryotherapy administration to maximize efficacy and tolerability of cryotherapy. Second, our sample size was calculated based on a pre-existing cryotherapy study (14, 30). However, the self-controlled design of that study might have amplified the effect of cryotherapy as their subjects were able to discern the difference in both the treated and untreated limbs. Thus, the actual benefit of cryotherapy could be much smaller than what was demonstrated.

With the randomized controlled study design, we generated robust evidence regarding the efficacy of cryotherapy supported with appropriate CIPN measures of PRO and electrophysiological assessments. PRO, though subjective, were designated as the primary outcome as they represent the direct impact of neuropathy on the lives and daily functioning of patients. Electrophysiological assessments objectively characterize the function of large and small nerve fibers after exposure to chemotherapy and mitigate the risk of placebo effect (48). The consistent results observed between these measures further substantiated our findings. The main limitation is the high rates of cryotherapy interruption which might have compromised the validity of our efficacy results. However, this was likely an accurate representation of patient behavior if cryotherapy were to be implemented in clinical practice. This emphasizes the need for future studies to improve the cryotherapy regimen before it can be introduced as a possible preventative strategy for CIPN.

## Conclusion

In our study, we were unable to conclude that cryotherapy prevents sensory neuropathy. While the greatest benefit of cryotherapy was observed at 3 months post-completion of paclitaxel treatment, it was not sustained in the long term which further limited the clinical significance of the intervention. This lack of efficacy may be attributable to the high rates of cryotherapy interruption. More research is necessary to optimize the cryotherapy regimen as cold intolerance remains an important factor in cryotherapy compliance. While results of this study dampen our initial enthusiasm of cryotherapy's role in preventing sensory symptoms related to CIPN, the observed effect of cryotherapy against small fiber neuropathy should be further explored with appropriate autonomic function and neuropathic pain outcome measures.

## Data Availability Statement

The original contributions presented in the study are included in the article and supplementary material, further inquiries can be directed to the corresponding authors.

## Ethics Statement

The studies involving human participants were reviewed and approved by SingHealth Centralized Institutional Review Board. The patients/participants provided their written informed consent to participate in this study.

## Author Contributions

AC and KL: conceived and designed the study. DN, CT, BS, MT, SL, YT, HO, PT, JC, WC, JL, GL, SB, TT, YY, GL, MW, RD, and KL: acquisition of data. DN, CT, YL, AC, and KL: analyzed and interpreted data. DN: drafted the manuscript. DN, CT, BS, MT, SL, YT, HO, PT, JC, WC, JL, GL, SB, TT, YY, GL, MW, RD, YL, AC, and KL: revised and approved the final version of the manuscript.

## Conflict of Interest

The authors declare that the research was conducted in the absence of any commercial or financial relationships that could be construed as a potential conflict of interest.
