# Supplementary material for: Impact of Cryotherapy on Sensory, Motor, and Autonomic Neuropathy in Breast Cancer Patients Receiving Paclitaxel: A Randomized, Controlled Trial
Source: Front Neurol. 2020 Dec 18;11:604688. doi: 10.3389/fneur.2020.604688 (PMC7793726; doi:10.3389/fneur.2020.604688)
Supplement: Supplementary file 1 [file Table_1.DOCX]

Supplementary Material

Impact of Cryotherapy on Sensory, Motor and Autonomic Neuropathy in Breast Cancer Patients Receiving Paclitaxel: A Randomized, Controlled Trial

# Supplementary Data

## Patient-reported outcome (PRO) measures

Three PRO questionnaires were used to assess chemotherapy-induced peripheral neuropathy (CIPN) symptoms and health-related QoL (HRQoL):

### Patient Neurotoxicity Questionnaire (PNQ)

PNQ is a two-item questionnaire measuring the severity of sensory and motor CIPN symptoms. Each item is graded from A (no neuropathy) to E (severe neuropathy) on a Likert scale. Convergent validity, discriminant validity and adequate responsiveness have been established in Japanese breast cancer patients who have received paclitaxel.(1,2) It is also supported by United States Food and Drug Administration as a clinical endpoint for the registration of neuroprotective drugs against CIPN.(3) Clinically important symptoms were determined as PNQ grades C-E as they delineate moderate to severe CIPN symptoms.

### European Organization for Research and Treatment of Cancer Quality of Life Questionnaire-CIPN twenty-item scale (EORTC QLQ-CIPN20)

EORTC QLQ-CIPN20 comprises twenty items that grade the degree of functioning affected by sensory, motor and autonomic CIPN symptoms.(4) Each item is graded numerically from 1 (not at all) to 4 (very much) on a Likert scale. It has been analyzed for internal consistency reliability, convergent validity, known-groups validity, structural validity and responsiveness.(5–7) In addition to subscale scores, a sum score of eighteen items, excluding conditional items 19 and 20, has been tested and recommended as a CIPN measure, whereby higher sum scores represent a greater degree of CIPN symptom burden.(6,8)

### EORTC Quality of Life Questionnaire-Core 30 (QLQ-C30) (version 3)

EORTC QLQ-C30 (version 3), the parent module of CIPN20, is a widely used to measure HRQoL.(9) There are thirty items, each graded 1 (not at all) to 4 (very much) on a Likert scale except for two global health status/QoL items which are graded from 1 (very poor) to 7 (excellent). Subscales for global health status (GHS), physical and role functioning, and pain symptoms were examined. Physical functioning (PF) measures the ability to perform physical activities while role functioning (RF) assesses the ability to carry out work, hobbies and other daily activities. Higher scores represent a better global health status and better degree of functioning while lower pain scores indicate less severe pain.(10)

# Supplementary Tables

**Table S1:** Mixed model analyses of NCS parameters at 1-2 weeks and 6 months post-paclitaxel treatment, with baseline adjustment (N=33)

|  | Amplitude | | | | | | Conduction Velocity | | | | | |
| --- | --- | --- | --- | --- | --- | --- | --- | --- | --- | --- | --- | --- |
|  | 1-2 weeks post-paclitaxel (T1) | | | 6 months post-paclitaxel (T3) | | | 1-2 weeks post-paclitaxel (T1) | | | 6 months post-paclitaxel (T3) | | |
|  | β | 95% CI | p-value | β | 95% CI | p-value | β | 95% CI | p-value | β | 95% CI | p-value |
| Sensory | |  |  |  |  |  |  |  |  |  |  |  |
|  | |  |  |  |  |  |  |  |  |  |  |  |
| Median | 2.141 | -4.072 – 8.355 | 0.499 | 0.411 | -7.391 – 8.213 | 0.918 | 2.889 | -0.548 – 6.326 | 0.099* | -1.477 | -5.979 – 3.024 | 0.520 |
| Ulnar | -2.722 | -7.565 – 2.12 | 0.271 | -2.792 | -8.734 – 3.15 | 0.357 | -0.389 | -3.276 – 2.498 | 0.792 | 0.074 | -2.632 – 2.779 | 0.958 |
| Radial | -1.488 | -5.692 – 2.715 | 0.488 | 0.636 | -4.771 – 6.042 | 0.818 | -1.007 | -3.242 – 1.229 | 0.377 | -2.25 | -6.097 – 1.597 | 0.252 |
| Peroneal | 0.719 | -0.819 – 2.257 | 0.359 | 0.518 | -1.181 – 2.216 | 0.550 | -0.352 | -8.428 – 7.723 | 0.932 | 3.178 | -9.534 – 15.89 | 0.624 |
| Sural | -0.164 | -3 – 2.672 | 0.910 | -1.037 | -2.632 – 0.557 | 0.202 | 0.575 | -8.671 – 9.821 | 0.903 | -6.51 | -14.93 – 1.913 | 0.130 |
| Motor |  |  |  |  |  |  |  |  |  |  |  |  |
|  |  |  |  |  |  |  |  |  |  |  |  |  |
| Median | -0.14 | -0.693 – 0.412 | 0.619 | -0.669 | -1.334 – -0.004 | 0.049* | 0.164 | -1.719 – 2.048 | 0.864 | -0.584 | -2.115 – 0.948 | 0.455 |
| Ulnar | -0.57 | -1.194 – 0.053 | 0.073 | -0.573 | -1.44 – 0.293 | 0.195 | 0.724 | -1.01 – 2.458 | 0.413 | -0.202 | -2.262 – 1.859 | 0.848 |
| Tibial | 1.354 | -0.348 – 3.057 | 0.119 | 0.559 | -1.017 – 2.135 | 0.487 | 0.015 | -3.376 – 3.406 | 0.993 | -0.903 | -3.258 – 1.452 | 0.452 |
| Peroneal | 0.34 | -0.295 – 0.974 | 0.294 | 0.269 | -0.207 – 0.744 | 0.269 | 0.173 | -1.733 – 2.079 | 0.859 | -1.552 | -3.022 – -0.083 | 0.038* |

Positive β values indicate better nerve function. *p<0.05.

**Table S2:** Mixed model analyses of changes in NCS parameters overtime, with baseline adjustment (N=33)

|  | Amplitude | | | | | | Conduction Velocity | | | | | |
| --- | --- | --- | --- | --- | --- | --- | --- | --- | --- | --- | --- | --- |
|  | Cryotherapy | | | Weeks (from baseline) | | | Cryotherapy | | | Weeks (from baseline) | | |
|  | β | 95% CI | p-value | β | 95% CI | p-value | β | 95% CI | p-value | β | 95% CI | p-value |
| Sensory | |  |  |  |  |  |  |  |  |  |  |  |
|  | |  |  |  |  |  |  |  |  |  |  |  |
| Median | 3.146 | -2.058 – 8.351 | 0.236 | -0.109 | -0.245 – 0.026 | 0.114 | 1.377 | -1.379 – 4.134 | 0.327 | -0.116 | -0.173 –  -0.059 | <0.001* |
| Ulnar | -2.122 | -6.424 – 2.181 | 0.334 | -0.006 | -0.108 – 0.096 | 0.909 | -0.308 | -2.188 – 0.019 | 0.748 | -0.03 | -0.078 – 0.019 | 0.232 |
| Radial | 0.046 | -4.461 – 4.552 | 0.984 | -0.036 | -0.116 – 0.043 | 0.373 | -1.954 | -4.147 – 0.239 | 0.081 | -0.035 | -0.084 – 0.014 | 0.157 |
| Peroneal | 0.375 | -1.055 – 1.805 | 0.607 | -0.089 | -0.12 –  -0.059 | <0.001* | 0.961 | -4.938 – 6.86 | 0.749 | -0.18 | -0.313 –  -0.047 | 0.008* |
| Sural | -0.783 | -2.94 – 1.373 | 0.477 | -0.101 | -0.139 –  -0.063 | <0.001* | -3.037 | -8.824 – 2.75 | 0.304 | -0.063 | -0.164 – 0.037 | 0.217 |
| Motor |  |  |  |  |  |  |  |  |  |  |  |  |
|  |  |  |  |  |  |  |  |  |  |  |  |  |
| Median | -0.276 | -0.745 – 0.193 | 0.249 | -0.002 | -0.012 – 0.008 | 0.708 | -0.052 | -1.364 – 1.26 | 0.938 | -0.026 | -0.055 – 0.004 | 0.090 |
| Ulnar | -0.639 | -1.35 – 0.072 | 0.078 | 0.01 | 0.001 – 0.019 | 0.032* | -0.062 | -1.363 – 1.24 | 0.926 | -0.032 | -0.062 –  -0.001 | 0.043* |
| Tibial | 0.821 | -0.504 – 2.147 | 0.225 | -0.032 | -0.052 –  -0.011 | 0.002* | 0.029 | -2.068 – 2.126 | 0.978 | -0.022 | -0.063 – 0.019 | 0.288 |
| Peroneal | 0.375 | -0.137 – 0.887 | 0.151 | 0.001 | -0.008 – 0.01 | 0.829 | -0.754 | -2.105 – 0.596 | 0.273 | -0.018 | -0.044 – 0.009 | 0.193 |

Positive β values indicate better nerve function. *p<0.05.

# References

1. Kuroi K, Shimozuma K, Ohashi Y, Hisamatsu K, Masuda N, Takeuchi A, et al. Prospective assessment of chemotherapy-induced peripheral neuropathy due to weekly paclitaxel in patients with advanced or metastatic breast cancer (CSP-HOR 02 study). Support Care Cancer. 2008/12/18. 2009;17(8):1071–80.

2. Shimozuma K, Ohashi Y, Takeuchi A, Aranishi T, Morita S, Kuroi K, et al. Feasibility and validity of the Patient Neurotoxicity Questionnaire during taxane chemotherapy in a phase III randomized trial in patients with breast cancer: N-SAS BC 02. Support Care Cancer. 2009;17(12):1483–91.

3. Hausheer FH, Schilsky RL, Bain S, Berghorn EJ, Lieberman F. Diagnosis, management, and evaluation of chemotherapy-induced peripheral neuropathy. Semin Oncol. 2006;33(1):15–49.

4. Postma TJ, Aaronson NK, Heimans JJ, Muller MJ, Hildebrand JG, Delattre JY, et al. The development of an EORTC quality of life questionnaire to assess chemotherapy-induced peripheral neuropathy: The QLQ-CIPN20. Eur J Cancer. 2005;41(8):1135–9.

5. Smith EML, Barton DL, Qin R, Steen PD, Aaronson NK, Loprinzi CL. Assessing patient-reported peripheral neuropathy: The reliability and validity of the European Organization for Research and Treatment of Cancer QLQ-CIPN20 Questionnaire. Qual Life Res. 2013;22(10):2787–99.

6. Kieffer JM, Postma TJ, van de Poll-Franse L, Mols F, Heimans JJ, Cavaletti G, et al. Evaluation of the psychometric properties of the EORTC chemotherapy-induced peripheral neuropathy questionnaire (QLQ-CIPN20). Qual Life Res. 2017;26(11):2999–3010.

7. Smith EML, Banerjee T, Yang JJ, Bridges CM, Alberti P, Sloan JA, et al. Psychometric Testing of the European Organisation for Research and Treatment of Cancer Quality of Life Questionnaire-Chemotherapy-Induced Peripheral Neuropathy 20-Item Scale Using Pooled Chemotherapy-Induced Peripheral Neuropathy Outcome Measures Standard. Cancer Nurs. 2019;42(3):179–89.

8. Smith EML, Knoerl R, Yang JJ, Kanzawa-Lee G, Lee D, Bridges CM. In Search of a Gold Standard Patient-Reported Outcome Measure for Use in Chemotherapy- Induced Peripheral Neuropathy Clinical Trials. Cancer Control. 2018;25(1).

9. Aaronson NK, Ahmedzai S, Bergman B, Bullinger M, Cull A, Duez NJ, et al. The European organization for research and treatment of cancer QLQ-C30: A quality-of-life instrument for use in international clinical trials in oncology. J Natl Cancer Inst. 1993;85(5):365–76.

10. Fayers P, Aaronson NK, Bjordal K, Groenvold M, Curran D, Bottomley A. The EORTC QLQ-C30 Scoring Manual (3rd Edition) [Internet]. The EORTC QLQ-C30 Scoring Manual (3rd Edition). European Organisation for Research and Treatment of Cancer, Brussels 2001; 2001. 1–67 p. Available from: http://www.eortc.be/qol/files/scmanualqlq-c30.pdf
